# β-Glucan-Producing *Pediococcus parvulus* 2.6: Test of Probiotic and Immunomodulatory Properties in Zebrafish Models

**DOI:** 10.3389/fmicb.2018.01684

**Published:** 2018-07-25

**Authors:** Adrián Pérez-Ramos, Maria L. Mohedano, Miguel Á. Pardo, Paloma López

**Affiliations:** ^1^Laboratory of Molecular Biology of Gram-positive Bacteria, Department of Microorganisms and Plant Biotechnology, Biological Research Center, Consejo Superior de Investigaciones Científicas, Madrid, Spain; ^2^Food Research Division, Centro Tecnológico de Investigación Marina y Alimentaria (AZTI), Derio, Spain

**Keywords:** *Pediococcus parvulus*, exopolysaccharides, β-glucans, lactic acid bacteria, probiotics

## Abstract

Lactic acid bacteria synthesize exopolysaccharides (EPS), which could benefit the host’s health as immunomodulators. Furthermore, EPS could protect bacteria against gastrointestinal stress, favoring gut colonization, thus protecting the host against pathogenic infections. *Pediococcus parvulus* 2.6, produces a 2-substituted (1,3)-β-D-glucan and, in this work, its probiotic properties as well as the immunomodulatory capability of its EPS have been investigated using *Danio rerio* (zebrafish). To this end and for a comparative analysis, *P. parvulus* 2.6 and its isogenic β-glucan-non-producing 2.6NR strain were fluorescently labeled by transfer of the pRCR12 plasmid, which encodes the mCherry protein. For the *in vivo* studies, there were used: (i) a gnotobiotic larvae zebrafish model for bacterial colonization, pathogen competition, and evaluation of the β-glucan immunomodulation capability and (ii) a transgenic (*mpx:GFP*) zebrafish model to determine the EPS influence in the recruitment of neutrophils under an induced inflammation. The results revealed a positive effect of the β-glucan on colonization of the zebrafish gut by *P. parvulus*, as well as in competition of the bacterium with the pathogen *Vibrio anguillarum* in this environment. The larvae treatment with the purified β-glucan resulted in a decrease of expression of genes encoding pro-inflammatory cytokines. Moreover, the β-glucan had an anti-inflammatory effect, when it was evaluated in an induced inflammation model of *Tg*(*mpx:GFP*) zebrafish. Therefore, *P. parvulus* 2.6 and its EPS showed positive health properties in *in vivo* fish models, supporting their potential usage in aquaculture.

## Introduction

Many lactic acid bacteria (LAB) reside in the intestinal tract of vertebrates. In addition, among these, it has been demonstrated that some strains belonging to the genera *Lactobacillus*, *Leuconostoc*, *Streptococcus*, and *Pediococcus* are probiotic, since they have beneficial effects for the health of the host organism (Guarner et al., 2017). The main mechanisms of action of probiotics are to strengthen the intestinal barrier, stimulate the immune system, or counteract the effects of pathogens through acidification of the environment, the production of antimicrobial substances, and competition for nutrients and adhesion to the epithelium (Guarner et al., 2017).

Many of these LAB produce a wide range of exopolysaccharides (EPS) that are considered prebiotic compounds (Salazar et al., 2016). These EPS could reach the colon where they are selectively fermented by the gut microbiota increasing beneficial anaerobic bacteria that contribute to the homeostasis and have beneficial effects on health (Verbeke et al., 2015). These bacteria and their EPS are used in the development of functional fermented foods (Zannini et al., 2016; Linares et al., 2017; Marco et al., 2017). Furthermore, the EPS, especially β-glucans, can act as immumodulators. β-Glucans are recognized by pattern recognition receptors such as dectin-1 and Toll-like receptors activating the NF-κB pathway that lead to the release of several cytokines (Chan et al., 2009; Lee and Kim, 2014). This activation stimulates the proliferation of monocytes and the maturation of dendritic cells and could be involved in anti-tumor responses (Meng et al., 2016).

*Pediococcus parvulus* 2.6 is a LAB isolated from Basque Country cider, whose genome has been determined (Pérez-Ramos et al., 2016). This bacterium produces a 2-substituted (1,3)-β-D-glucan of high molecular mass (Dueñas-Chasco et al., 1997). Evaluation of this bacterium using *in vitro* models showed the influence of β-glucan in some probiotic properties such as adhesion to human enterocyte cell lines and immunomodulation of macrophages (Fernández de Palencia et al., 2009). This β-glucan provided enhancement of bacterial growth and adhesion capability of lactobacilli (Russo et al., 2012), and was also able to activate human macrophages with an anti-inflammatory response (Notararigo et al., 2014). Furthermore, a *P. parvulus* 2.6 fermented cereal-based matrix, producing the β-glucan *in situ* resulted in improved technological and functional features of the products (Pérez-Ramos et al., 2017a). Moreover, these β-glucan enriched matrices have a protective effect and improve the growth of probiotic LAB (Pérez-Ramos et al., 2017a). Thus, the previous works support the potential of the *P. parvulus* 2.6 strain as a component of functional human food. In addition, we have previously shown that EPS-producing LAB isolated from meat fermented products have potential as fish probiotics and its EPS as antimicrobial and immunomodulator (Nácher-Vázquez et al., 2015, 2017). Therefore, *P. parvulus* 2.6 strain could also have interest for the fish feed sector.

Fish is a good food for humans, because its proteins are highly digestible, and it contains vitamins and minerals, and is the most important source of ω3 lipids, which are important for reducing cardiovascular risk (Marik and Varon, 2009). Wild fish is a limited resource on earth, but fish farms could assure the human supply in the future. To increase the profitability of aquaculture, one of the most important aspects is to reduce the cost of feeding. For this aim, in recent years, the use of feed with 100% vegetable proteins has obtained successful results. However, the use of these kinds of feeds present certain problems related to the fish immune system, affecting their intestinal morphology and producing diseases such as enteritis and diarrhea. These pathological problems can lead to death due to bacterial or viral outbreaks, which can cause considerable economic loss in aquaculture (Subasinghe, 2005; Torrecillas et al., 2007). To diminish this problem, β-glucans from plant, fungi, and yeast are widely utilized to avoid the outbreak of infectious diseases (Meena et al., 2013). Thus, the β-glucan produced by *P. parvulus* 2.6 as well as the bacterium itself could be of interest in aquaculture as an immunomodulant and as a probiotic, respectively. To prove these potential beneficial effects, its test in animal models is required. To this end, the zebrafish model has become a powerful tool for the study of vertebrate development, immunity, and diseases (Phelps and Neely, 2005; Goldsmith and Jobin, 2012; Novoa and Figueras, 2012). This is due to its high fecundity, small size, optical transparency at the beginning of the developmental stages, availability of genetic tools, and its immune system that possesses a high similarity to that of other vertebrates. Moreover, gnotobiotic zebrafish larvae are relatively easy to obtain and provide a good model to study bacterial colonization (Rendueles et al., 2012; Rieu et al., 2014; Russo et al., 2015) or host–pathogen interaction (Oyarbide et al., 2015) without interference of the gut microbiota or environmental microorganisms. In addition, the optical transparency of zebrafish larvae allows the easy detection of fluorescent-labeled bacteria for studies of host–microbe interactions (Rieu et al., 2014; Oyarbide et al., 2015; Russo et al., 2015). Thus, the development of fluorescence reporter systems to label putative probiotic bacteria is useful for tracking them inside the animal model used (van Zyl et al., 2015; Landete et al., 2016). Accordingly, we have developed the pRCR12 plasmid (Russo et al., 2015) derived from the pRCR promoter probe vector (Mohedano et al., 2015), which expresses constitutively the red fluorescent protein mCherry, it is useful for the fluorescent-labeling and detection of LAB, and it has been already validated in *Lactobacillus acidophilus*, *Lactobacillus casei*, *Lactobacilus fermentum*, *Lactobacillus plantarum*, *Lactobacillus sakei*, *Lactococcus lactis*, and *Streptococcus pneumoniae* (Mohedano et al., 2015; Russo et al., 2015; Nácher-Vázquez et al., 2017; Pérez-Ramos et al., 2017b). Moreover, the fluorescence labeling of *L. fermentum*, *L plantarum*, and *L. sakei* with pRCR12 has allowed visualization of these bacteria in the zebrafish gut (Russo et al., 2015; Nácher-Vázquez et al., 2017).

Thus, in this work, we have labeled *P. parvulus* 2.6 and its isogenic EPS-non-producing 2.6NR strains with the pRCR12 plasmid and zebrafish larvae models were used to perform *in vivo* a comparative study of the probiotic properties of these strains as well as of the immunomodulatory properties of the β-glucan produced by *P. parvulus* 2.6.

## Materials and Methods

### Bacterial Strains, Growth Conditions, and Plasmid

The 2-substituted (1,3)-β-D-glucan-producing *P. parvulus* 2.6 and its isogenic non-producing strain *P. parvulus* 2.6NR (Fernández et al., 1995) were used in this work. *L. plantarum* 90[pRCR12] (Russo et al., 2015) was used as source of plasmid pRCR12 to electrotransform the *Pediococcus* strains. Plasmid pRCR12 carries a transcriptional fusion of the pneumococcal P_x_ promoter and the *mrfp* gene, whose codons are optimized for expression in LAB, and which encodes a monomeric version of the red fluorescent protein of *Discosoma* sp. (Garcia-Cayuela et al., 2012). LAB were routinely grown at 30°C in Man Ragosa Sharpe (MRS) medium (Pronadisa, Spain) supplemented with chloramphenicol (Cm) at 10 μg mL^-1^ for growth of strains carrying pRCR12 plasmid. *Escherichia coli* V517 is a multiple plasmid strain (Macrina et al., 1978) used in this work as standard to determine molecular weight of plasmids, and it was grown in LB broth (Pronadisa, Spain) at 37°C. The *Vibrio anguillarum* NB10[pOT11] serotype O1 strain used in this work (O’Toole et al., 2004) was kindly provided by R. O’Toole from Umeå University. This is a green fluorescent protein (GFP)-labeled bacterium due to the expression of the inducible *tac-gfpmut2* transcriptional fusion carried by the pOT11 plasmid. The bacterium was grown at 25°C in TSB (tryptic soy broth, Pronadisa) supplemented with Cm at 10 μg mL^-1^ and 0.5 mM isopropyl-β-D-thiogalactopyranoside to induce expression of the GFP protein.

### Antibiotic Resistance Profile of *P. parvulus* 2.6 and 2.6NR Strains

The bacteria were screened for antibiotics resistance. The antibiotics recommended by the European Food Safety Authority (EFSA, 2012) to identify bacterial strains with potential acquired resistance to antibiotics were analyzed. The antibiotics tested were: ampicillin, chloramphenicol, clindamycin, erythromycin, gentamycin, kanamycin, streptomycin, tetracycline, and vancomycin. The minimal inhibitory concentration (MIC) was determined by the broth microdilution method reported by the ISO 10,932/IDF 233 standard (International Organization for Standardization, 2010). The strains were classified as susceptible or resistant according to the cut-off values proposed by EFSA (2012). A bacterial strain was defined as susceptible when its growth was inhibited at a specific antimicrobial concentration equal or lower than the established cut-off value and it was considered resistant when its growth was not inhibited at a concentration higher than the established cut-off value.

### pRCR12 DNA Preparation and Transfer to *P. parvulus* Strains

The pRCR12 plasmid from *L. plantarum* 90 was isolated using the high pure plasmid isolation kit (Roche) as follows. The strain was grown to stationary phase [10^9^ colony forming units (cfu) mL^-1^] and 1 mL of the culture was sedimented by centrifugation at 10,000 × *g* for 10 min at 4°C. The cells were resuspended in solution I of the kit supplemented with lysozyme (30 mg mL^-1^) and were incubated for 30 min at 37°C. Then, plasmid isolation was performed as described in the kit protocol, eluting the plasmidic DNA in 100 μL at approximately 100 ng μL^-1^.

*P. parvulus* 2.6 and 2.6NR strains were electrotransformed with pRCR12 using the method of Berthier et al. (1996) with modifications, as follows. Bacterial cultures were grown in MRS supplemented with 40 mM D-threonine to an optical density at 600 nm (OD_600 nm_) of 0.8 (3 × 10^8^ cfu mL^-1^), sedimented by centrifugation at 5,500 × *g* for 10 min at 4°C, and the cells subjected to two cycles of resuspension in phosphate buffered saline (PBS) solution at pH 7.2 and sedimentation as above. In the case of the 2.6 strain, the bacteria were thoroughly vortexed during resuspension to remove its β-glucan from the bacterial surface to facilitate the subsequent incorporation of plasmidic DNA. Then, the cells were resuspended in 1 mL of lysozyme solution (final concentration of 2,000 U mL^-1^) in PBS pH 7.2 and incubated for 20 min at 37°C. Afterward, bacterial cells were sedimented (3,300 × *g*, 5 min, 4°C) and subjected to cycles of resuspension in 1 mL (once in PBS pH 7.2, twice in 10 mM MgCl2) and once in electroporation buffer (0.5 M sucrose plus 10% glycerol) and sedimentation (3,300 × *g*, 5 min, 4°C), and the bacteria were resuspended in the electroporation buffer to a concentration of about 4 × 10^10^ cfu mL^-1^. Then, 5 μL of pRCR12 plasmid (0.5 μg) was added to 50 μL of the bacterial suspension and were electroporated at 1.8 kV, 600 Ω, and 25 μF in a 0.2-cm cuvette using a Gene Pulser Xcell with ShockPod cuvette chamber (Bio-Rad, Hercules, CA, United States), obtaining a time constant of 12.5–13 ms. Transformants were selected in MRS-agar supplemented with Cm at 10 μg mL^-1^. The obtained recombinant strains were designated 2.6[pRCR12] and 2.6NR[pRCR12].

### Growth and Fluorescence Analysis of *P. parvulus* Strains 2.6[pRCR12] and 2.6NR[pRCR12]

Growth and red fluorescence emitted by the *P. parvulus* strains were monitored simultaneously with the Varioskan Flask System (Thermo Fisher Scientific, Waltham, MA, United States), which provides quantitative data of cell density by measuring the OD_600 nm_ and mCherry expression upon excitation at a wavelength of 587 nm and detection of emission at a wavelength of 612 nm. Overnight cultures were diluted in fresh medium to give an OD_600 nm_ = 0.1 and 300 μL of each culture was placed in triplicated in a sterile 96-Well Optical White w/Lid Cell Culture (Thermo Fisher Scientific, Rochester, NY, United States). The experiments were performed in triplicate incubating in the Varioskan at 30°C and measuring OD and fluorescence at 30 min intervals.

### Fluorescence Microscopy and Transmission Electron Microscopy

Exponential cultures of *P. parvulus* strains and *V. anguillarum* NB10[pOT11] were sedimented and concentrated fivefold by resuspension in PBS pH 7.2. Then, without fixing, the suspension (10 μL) was used for phase contrast and fluorescent microscopy analysis with a Leica DM1000 model microscope (Leica Microsystems, Mannheim, Germany) with a light source EL6000 and the filter system TX2 ET and GFP ET for detection of mCherry and GFP fluorescences, respectively. The microscope was connected to a DFC3000G camera (Leica Microsystems) with a CCD sensor. The image analysis was performed using Leica Application Suite X Software (Leica Microsystems).

Cultures of 2.6[pRCR12] and 2.6NR[pRCR12], prepared as described for binding to Caco-2 cells assay (see below), were used for electron microscopy analysis. A drop from each bacterial solution resuspended in 0.1 M AcNH_4_, pH 7, was deposited on a carbon film copper grid, which had previously been hydrophilized by a glow discharge process for 1 min, and rinsed in water during 15 s. Then, the grid was stained during 10 s with a uranyl acetate water solution (0.2% w/v) in order to improve the image contrast, and finally, rinsed again in water. The sample was air-dried and examined in a JEOL JEM-1230 microscope (JEOL, Peabody, MA, United States), operating at an accelerating voltage of 100 kV.

### Production, Purification, and Labeling of the 2-Substituted (1,3)-β-D-Glucan

The β-glucan of *P. parvulus* 2.6 was produced and purified by ethanol precipitation, dialysis, and chromatographic fractionation as previously described (Notararigo et al., 2013). 5-([4,6-Dichlorotriazin-2-yl] amino fluorescein hydrochloride (DTAF; Sigma-Aldrich) was used to fluorescently label the β-glucan; 1.6 mg of DTAF and 4 mg of β-glucan were dissolved in 2 mL of 0.1 M borate buffer, pH 9. The mix was incubated for 16 h at 25°C under an agitation of 600 rpm. The solution was dialyzed for 1 day against distilled water (changed twice), using a dialysis membrane having a cut-off of 12–14 kDa. After dialysis, the solution was frozen at -80°C, lyophilized, and kept at room temperature until its use for detection in zebrafish gut.

### Quantification of the 2-Substituted (1,3)-β-D-Glucan

To determine the total concentration of the β-glucan produced by *P. parvulus* strains, bacteria were grown in MRS medium until late exponential phase (OD_600 nm_ = 3.0). Then, after vortexing to release the β-glucan attached to the bacteria, the bacteria were sedimented by centrifugation (16,000 × *g*, 10 min, 4°C), and the β-glucan present in the supernatants was quantified using a competition ELISA method (Werning et al., 2014). This method is based on the *S. pneumoniae* serotype 37 antibodies that can recognize specifically this bacterial β-glucan. The purified β-glucan of *P. parvulus* 2.6 was immobilized in each well of a 96-Well Nunc-Immuno MicroWell MaxiSorp plate (Thermo Fisher Scientific, Roskilde, Denmark) and used to compete with the β-glucan presented in the culture supernatants for binding to the primary antibody (antiserotype 37, Statens Serum Institut, Copenhagen, Denmark). The whole assay was carried out as previously described (Werning et al., 2014).

The ELISA method was also used to determine the concentration of β-glucan attached to the producing bacteria, with or without its removal, that were used for the adhesion assays to Caco-2 cells (see details above). Bacteria grown and treated as described above were resuspended in PBS pH 7.2 prior to direct determination of β-glucan concentration.

### Plasmid Analysis and Detection of the *gtf* Gene by Southern Hybridization

With the aim of confirming the lack of plasmid rearrangements after transfer of pRCR12, the total plasmidic DNA of the *P. parvulus* parental and recombinant strains was obtained and location of the *gtf* genes was analyzed by Southern blot hybridization. Total plasmid DNA preparations of *P. parvulus* strains were obtained and purified by isopycnic CsCl density gradient to eliminate non-supercoiled forms of the plasmids as previously described (Pérez-Ramos et al., 2017b). Plasmid samples were fractionated in a 0.7% agarose gel and DNA molecules were revealed by staining with ethidium bromide at 0.5 mg mL^-1^. The images of the gel were obtained with the GelDoc 200 equipment and the Quantity one 4.5.2 software (Bio-Rad, Laboratories Ltd., Alcobendas, Spain). For Southern blot hybridization, the DNA fragments were transferred to a nylon membrane as previously described (Pérez-Ramos et al., 2017b). An internal region of 598 bp of the *gtf* gene was amplified as previously described (Werning et al., 2006) by PCR with primers GTFSF (5′-TTGCCAGAACTAGAGAAAGTACGCA-3′) and GTFSR (5′-ACTTCCTATTTTAGCTAAAAAGCAA-3′) using as substrate a total plasmidic DNA preparation of *P. parvulus* 2.6. The amplicon was labeled with digoxigenin-dUTP by using the DIG high prime DNA labeling and detection starter kit II (Roche, Mannheim, Germany) and the hybridization was performed as previously described (Pérez-Ramos et al., 2017b). The signals were detected with the LAS-3000 imaging system (Fujifilm, Stamford, CT, United States).

### Adhesion of *P. parvulus* Strains to Caco-2 Cells

The Caco-2 human enterocyte cell line, obtained from the cell bank at CIB, was seeded in 96-well tissue culture plates (Falcon Microtest^TM^, Becton Dickinson, Franklin Lakes, NJ, United States) at a final concentration of 1.25 × 10^5^ cells mL^-1^ and grown as mono-layers of differentiated cells for 14 days as previously described (Nácher-Vázquez et al., 2017). Cell concentrations were determined as previously described (Garai-Ibabe et al., 2010).

To test the adhesion, overnight cultures of *P. parvulus* strains were diluted to give an OD_600 nm_ = 0.1 at 30°C and grown for 20 h, until late exponential phase. Then, samples of the *Pediococcus* strains were sedimented by centrifugation (9,300 × *g*, 10 min, 4°C), gently resuspended in PBS pH 7.2, and sedimented as before. In addition, with the aim of removing the EPS present on the surface of the *P. parvulus* 2.6[pRCR12] cells, a fraction of its bacterial culture designated 2.6p^∗^ was thoroughly vortexed prior to sedimentation as above and further subjected to two cycles of resuspension in PBS and sedimentation. Afterward, the bacterial samples were resuspended in Dulbecco’s Modified Eagle Medium (DMEM, Invitrogen) at a concentration of 1.25 × 10^6^ cfu mL^-1^ and added to the Caco-2 cells in a final volume of 0.1 mL per well. After incubation for 1 h at 37°C in an atmosphere containing 5% CO_2_, unattached bacteria were removed by three washing with 0.2 mL of PBS pH 7.2. Then, Caco-2 cells were detached from the well by incubating with 0.1 mL of 0.05% (w/v) trypsin-EDTA for 5 min at 37°C. The reaction was stopped by addition of 0.3 mL of PBS pH 7.2. To determine the number of cell-associated bacteria, appropriate dilutions were plated onto MRS-agar supplemented with Cm at 10 μg mL^-1^. Three independent adhesion assays were performed in duplicate.

### Animal Husbandry

Zebrafish embryos were obtained from wild-type adult zebrafish (*Danio rerio*, Hamilton, 1822), which were bred and maintained in the AZTI Zebrafish Facility (REGA number ES489010006105; Derio, Spain) as previously described (Russo et al., 2015) following standard conditions (Sullivan and Kim, 2008). During experimentation, the fish were maintained at an average density of 1.3 animals per mL in sterile petri dishes housed in an air incubator at 27°C on a 12 h light cycle. All experimental procedures were approved by the Regional Animal Welfare Body (Project ENVIPHAGE, NEIKER-OEBA-2015-004).

Production of germ-free zebrafish was performed as previously described (Oyarbide et al., 2015). Briefly, embryos were washed with a sterilized embryo wash buffer (EWB) solution [embryo water (EW) solution (CaCl_2_ at 220.5 mg L^-1^, MgSO_4_ 7 H_2_O at 92.5 mg L^-1^, NaHCO_3_ at 47.3 mg L^-1^, and KCl at 4.1 mg L^-1^) supplemented with methylene blue 0.01% (w/v)], antibiotic solution (kanamycin 15 μg mL^-1^, ampicillin 300 μg mL^-1^, and amphotericin B 1.25 μg mL^-1^), and 0.02% (w/v) polyvinylpyrrolidone for 2 min. Then, washed with 0.003% (v/v) bleach solution for 1 h and finally washed with EWB solution. Afterward, the embryos were incubated overnight in antibiotic solution. The following day 50 embryos were collected and transferred to a Petri dish (5.5 cm diameter × 1.0 cm) to be immersed in 5 mL EWB solution and treated with two UV light pulses of 1.6 kV (Pulsed UV System XeMatica 1:2L-SA, SteriBeam Systems, GmbH) to inactivate bacteria present in the sample. The entire procedure was carried out inside a laminar flow cabinet to maintain sterile conditions; sterile solutions and materials were also used. Sterility was routinely tested after four days post fertilization (dpf), by culturing on general aerobic and yeast and molds culture media (Petrifilm aerobic, and Petrifilm yeast and molds count plates, 3 M).

### Zebrafish Gut Colonization by *P. parvulus* 2.6[pRCR12] and 2.6NR[pRCR12] Strains

Cultures of *P. parvulus* strains were grown and treated in the same way as for the Caco-2 cells adhesion assays. Groups of 40 gnotobiotic zebrafish larvae at 4 dpf were placed into each Petri dish and were incubated with 30 mL of EW solution containing one of the bacterial strains at 5 × 10^7^ cfu mL^-1^. Larvae were incubated at 28°C with agitation (60 rpm) for 18 h. Then, the larvae were transferred to other sterile Petri dishes and the bacteria adhered to their surface were eliminated by washing them five times with PBS pH 7.2, and maintained in 30 mL of the same buffer. After, 6, 24, and 48 h post exposure (hpe), larvae were euthanized with tricaine at 300 mg mL^-1^, and were individually visualized using a Leica MZFL III stereomicroscope (LeicaMicrosystems GmbH, Wetzlar, Germany) with a zoom magnification range of 8× to 100×. The microscope was equipped with a visible light and an ultraviolet light (Hg 100 W) source. mCherry fluorescence was detected by exposure of the larvae to ultraviolet light in the excitation range of 540–580 nm. Images were captured using a Leica DFC 360FX camera and processed with the LAS-AF (Leica Microsystems GmbH). In addition, three pools of five larvae of each treatment were washed twice in PBS containing 0.1% tween 20 to remove bacteria loosely attached to the skin, once with PBS pH 7.2, and finally were resuspended in 0.4 mL of the same buffer. Then, larvae were mechanically homogenized with a Pellet Pestle Cordless Motor (Kimble Chase Life Science and Research Products LLC), and the solutions obtained were plated onto MRS supplemented with Cm 10 μg mL^-1^ plates. Bacterial cfu were counted after incubation at 30°C for 48 h. Three independent experiments were performed.

### *P. parvulus* 2.6[pRCR12] and 2.6NR[pRCR12] Strains Activity Against *V. angillarum* NB10[pOT11] in the Zebrafish Model

Groups of 20 gnotobiotic zebrafish larvae at 4 dpf were pre-treated with either 2.6[pRCR12] or 2.6NR[pRCR12] strains as described above for 18 h. Then, the larvae were infected with bacterial solutions of *V. anguillarum* NB10[pOT11] previously grown during 16 h, sedimented by centrifugation (9,300 × *g*, 10 min, 4°C), and subjected to two cycles of resuspension in EW solution containing 0.5% NaCl and sedimentation as before. Finally, they were resuspended in EW to give a solution of 10^8^ cfu mL^-1^. Also, 20 gnotobiotic zebrafish larvae were infected with *V. anguillarum* without bacterial pre-treatment, and used as a positive infection control. In addition, 10 gnotobiotic zebrafish larvae were incubated in the absence of LAB and *V. anguillarum*, and used as a control of lack of mortality in the absence of infection. Larvae were individually placed into wells of 24-well plates with 1 mL of *V. anguillarum* solution. Bacterial solution was changed every 24 h and the infection was extended for 72 h. Mortality was examined at 24, 48, and 72 h post infection (hpi). Three independent experiments were performed in triplicate.

### Antimicrobial Activity of *P. parvulus* Strains Against *V. anguillarum* Tested *in Vitro*

An agar spot test and a well diffusion assay were used, according to Schillinger and Lucke (1989). For the spot test, overnight cultures of *Pediococcus* strains were spotted (5 μL) onto the surface of MRS-agar plates, and incubated for 30 h at 30°C to allow colonies to develop. Approximately 5 × 10^7^ CFU of *V. anguillarum* were inoculated into 10 mL of TSB-agar (containing 1% agar) and poured over the plates on which *Pediococcus* strains were grown. The plates were incubated for 24 h at 25°C and checked for inhibition zones. For the well diffusion assay, overnight cultures of *Pedioccocus* strains were sedimented by centrifugation (10,000 × *g*, 15 min at 4°C). Supernatants were recovered and the pH was measured. Then, an aliquot of the supernatants was adjusted to pH 6.5 with 3 M NaOH and sterilized by 0.22 μm pore-size Minisart^®^ syringe filters (Sartorius stedim biotech, Gotinga, Germany). Approximately 5 × 10^7^ CFU of *V. anguillarum* were inoculated into 10 mL of TSB-agar and poured over sterile plates. After solidification, wells of 3 mm of diameter were made in the agar plates; 60 μL of the supernatants with pH adjusted, or without, were placed into the wells; 0.1 M HCl and MRS medium were used as positive and negative controls, respectively. The plates were left at 4°C for 2 h to allow diffusion of the tested supernatants and then incubated for 24 h at 25°C. The absence and presence of inhibitory zones around the wells were recorded.

### Immunomodulation of Gnotobiotic Zebrafish Larvae by the *P. parvulus* 2.6 β-Glucan

To show if the β-glucan enters inside of the zebrafish gut, five gnotobiotic larvae at 4 dpf were submerged in a solution of DTAF-labeled β-glucan at 150 μg mL^-1^ during 16 h. Then, the larvae were washed and after 6 h they were visualized using the Leica MZFL III stereomicroscope and the DTAF fluorescence was detected by exposure of the larvae to UV light in the excitation range of 450–490 nm. For the immunomodulation assay, groups of 20 gnotobiotic zebrafish larvae at 4 dpf were submerged in EW containing β-glucan at 150 μg mL^-1^ (treated) or in EW (control) solutions during 30 h. Then larvae were frozen in liquid nitrogen and total RNA was extracted using Trizol Reagent (Invitrogen Life Technology, Merelbeke, Belgium) according to the manufacturer’s instructions. The quantity and quality of RNA samples were determined by capillary electrophoresis, using an Agilent 2100 Bioanalyzer (Agilent Technologies, las Rozas, Spain); 20 ng of each RNA sample was used to synthesize cDNA with the oligo d(T)_16_ in a reverse transcription reaction with the TaqMan^®^ Reverse Transcription kit (Applied Biosystems Life Technology, Belgium) and following the instructions of the manufacturer. Changes in mRNA expression of several genes, related to the innate immune system, were monitored by real-time qPCR, performed with SYBR Green PCR master mix (Roche Diagnostics, Rotkreuz, Switzerland) on a Roche LightCycler^®^ 96 Instrument. The gene tested and the sequences of the primers used are listed in **Table 1**. The reaction conditions were: 95°C for 10 min followed by 40 cycles of 95°C for 10 s and 60°C for 30 s, and a dissociation step of 95°C for 1 min, 65°C for 1 min, and 95°C for 15 s. The mean *C*_T_ of each sample was normalized against the housekeeping genes (β-actin and elongation factor 1) and the corresponding control. The relative quantification of each gene was calculated by the 2^-ΔΔCT^ method, using the REST 2009 software (Qiagen, Hilden, Germany).

**Table 1:** Oligonucleotides used in qPCR reactions.

Primers	Sequence (5′–3′)	Gene	NCBI ID
ACT-F	TGCTGTTTTCCCCTCCATTG	Beta actin	NM_131031.1
ACT-R	TTCTGTCCCATGCCAACCA		
EF1-F	GCCAACTTCAACGCTCAGG	Elongation factor 1	NM_131263.1
EF1-R	AGAGATCTGACCAGGGTGGTTC		
IL1B-F	CATTTGCAGGCCGTCACA	Interleukin 1-β	NM_212844.2
IL1B-R	GGACATGCTGAAGCGCACTT		
IL8-F	CCCTCTGCTCCATGGGTTAA	Interleukin 8 (Chemokine 8)	XM_001342570.3
IL8-R	CAGGTGATCCGGGCATTC		
MyD88-F	CACAGGAGAGAGAAGGAGTCACG	Myeloid differentiation primary response gen 88	NM_212814.2
MyD88-R	ACTCTGACAGTAGCAGATGAAAGCAT		
NFKB-F	AGAGAGCGCTTGCGTCCTT	Nuclear factor kappa B	NM_001003414.1
NFKB-R	TTGCCTTTGGTTTTTCGGTAA		
TLR2-F	GGAAGGTGGCACTAAGAGCCT	Toll-like receptor 2	NM_212812.1
TLR2-R	TGATCGGTCGTGGAGGAGTT		
TLR22-F	CCAGCTCTCGCCGTACCA	Toll-like receptor 22	NM_001128675.2
TLR22-R	TTGGGCCAGCGGATGT		
TLR4-F	GGGAAGTCAATCGCCTCCA	Toll-like receptor 4	NM_001121051.1
TLR4-R	ACGGCTGCCCATTATTCCT		
IL10-F	ATATTTCAGGAACTCAAGCGGG	Interleukin 10	NM_001020785
IL10-R	ACTTCAAAGGGATTTTGGCAAG		
TNFa-F	ACCAGGCCTTTTCTTCAGGT	Tumor necrosis factor alpha	NM_212859.2
TNFa-R	GCATGGCTCATAAGCACTTGTT		


### Evaluation of *P. parvulus* 2.6 β-Glucan in a Model of Induced Inflammation of Zebrafish

The zebrafish transgenic line *Tg*(*mpx:GFP*)*i114* (Renshaw et al., 2006) whose neutrophils are GFP-labeled was used to make an induced inflammation model. β-glucan of the 2.6 strain purified as described in Section “Production, Purification, and Labeling of the 2-Substituted (1,3)-β-d-Glucan” was dissolved in EW solution at 150 μg mL^-1^. The inflammation was induced on 14 larvae at 3 dpf by cutting off the apical region of their tails. Then, the larvae were divided into two groups and immediately individually submerged in EW containing β-glucan (treated) or in EW (control) solutions. Fluorescence images of each larva were taken at 0, 4, 8, and 24 h using the Leica MZFL III stereomicroscope and the GFP fluorescence was detected as indicated above for DTAF. The Images were processed and analyzed using the ImageJ 1.51w software (National Institute of Health, Maryland, United States). Three independent experiments were performed.

### Statistical Analysis

In the Caco-2 cells adhesion assays as well as gut colonization and *Vibrio* infection analyses, the data are expressed as mean ± SD calculated from three independent experiments. The data were subjected to one-way analysis of variance (ANOVA) by using the SAS software. Tukey’s test was employed to determine the significant differences between the variables at *p* ≤ 0.05. In the induced inflammation analysis, the data are expressed in logarithms as a mean ± SD calculated from three independent experiments. The two-sample *t*-test was employed to determine the significant differences between the control group and the treated group at *p* ≤ 0.05.

## Results

### Construction and *in Vitro* Characterization of *P. parvulus* 2.6[pRCR12] and 2.6NR[pRCR12] Strains

**FIGURE 1:**
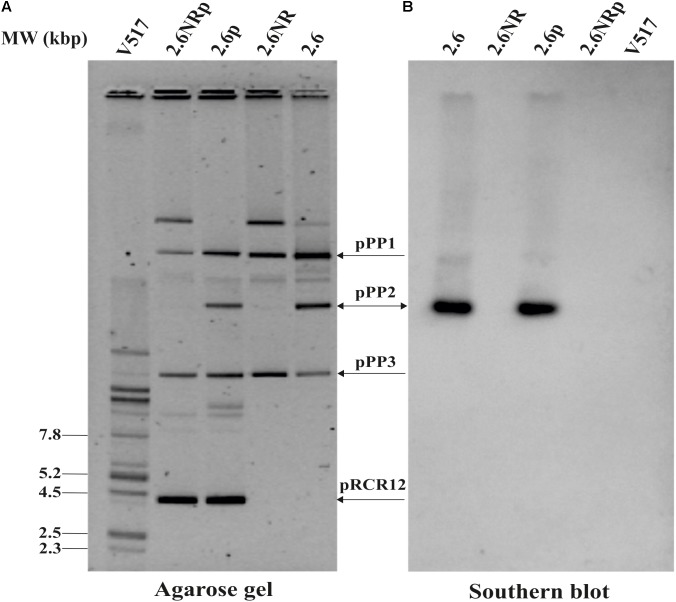
Detection of *P. parvulus* plasmids and *gtf* gene. Plasmids’ preparations of *P. parvulus* 2.6, 2.6NR, 2.6p, and 2.6NRp strains and of *E. coli* V517 were fractionated by electrophoresis in a 0.7% agarose gel, transferred to a nylon membrane, and the *gtf* gene detected by Southern blot hybridization. **(A)** The gel. **(B)** The hybridized membrane. The headed and double headed arrows indicate the positions of the covalently closed monomeric forms of the plasmids and the position of the hybridized bands in the corresponding gel, respectively.

As stated above, previous results indicated the probiotic potential of the 2-substituted (1,3)-β-D-glucan-producing *P. parvulus* 2.6 (reviewed in Pérez-Ramos et al., 2015). In addition, evaluation of this bacterium and its isogenic non-producing 2.6NR strain according with the EFSA specifications revealed that these bacteria were sensitive to all the antibiotics tested (Supplementary Table S1), and consequently there were not substrates for horizontal transfer of antibiotic resistance determinants. Therefore, we envisaged the *in vivo* evaluation of these pediococci in zebrafish models. Thus, with the aim of visualizing pediococci in the gut of the zebrafish larvae, the 2.6 and 2.6NR strains were fluorescently labeled with the mCherry protein by transfer of the pRCR12 plasmid. The resulting recombinant strains were designated 2.6[pRCR12] (2.6p) and 2.6NR[pRCR12] (2.6NRp) and total plasmid DNA preparations of these strains were compared with those of the parental strains by analysis in agarose gels (**Figure 1**) to confirm that the genetic manipulations have not affected the former plasmid content. *P. parvulus* 2.6 carries three natural plasmids: pPP1 (39.1 kbp), pPP2 (24.5 kbp), and pPP3 (12.7 kbp; Pérez-Ramos et al., 2017b), and the 2.6NR strain was generated from 2.6 by pPP2 plasmid curing (Fernández et al., 1995). Thus, according with these differences, the plasmidic DNA analysis revealed that 2.6p and 2.6NRp harbor the expected plasmids and that these bacteria also carry the 4.4 kbp pRCR12 plasmid. In addition, the plasmidic profiles showed that the establishment of pRCR12 in these hosts did not modify significantly the ratios of the natural plasmids. Consequently, it seems that there is no incompatibility between the replication machinery of pRCR12 plasmid and those of the other plasmids. The pPP2 plasmid harbors the *gtf* gene, which encodes the GTF glycosyltransferase responsible for the β-glucan synthesis (Werning et al., 2006). Therefore, we searched for the presence of the *gtf* gene in the plasmidic DNA samples. A Southern blot hybridization of the total plasmidic DNA preparations of the four *P. parvulus* strains was performed using an internal region of the *gtf* gene as a probe. As expected, hybridization signals were observed at the position of the 24.5 kbp pPP2 plasmid only in the 2.6 and 2.6p DNA samples (**Figure 1**).

**FIGURE 2:**
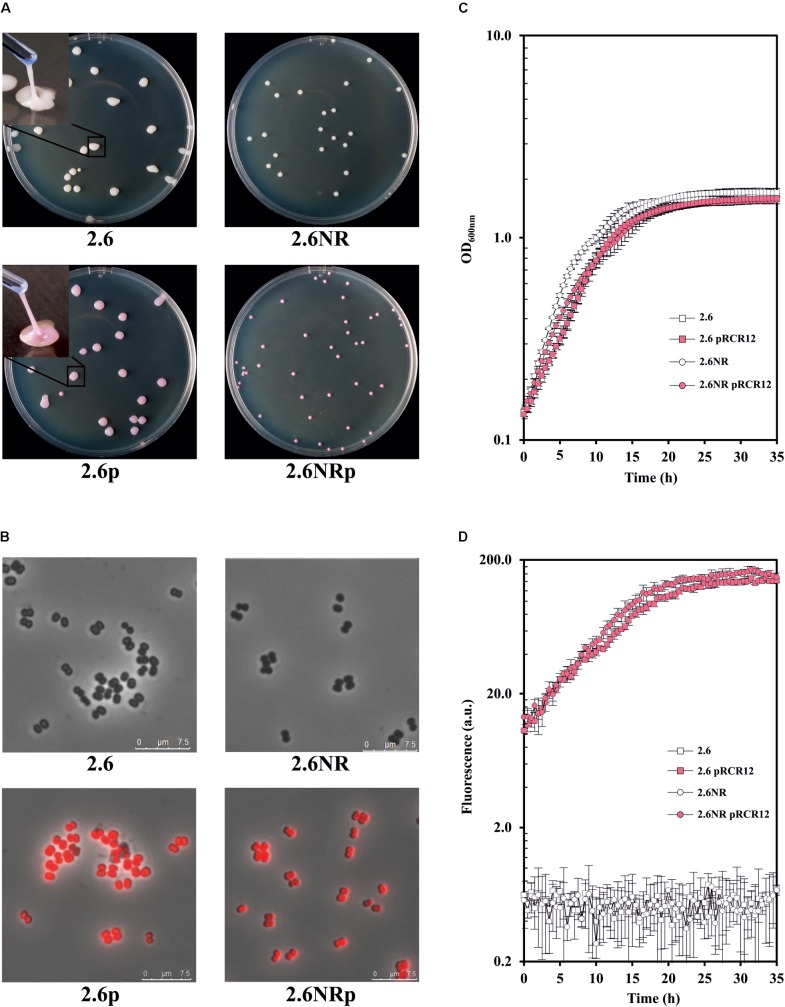
Comparative analysis of the *P. parvulus* 2.6, 2.6NR, 2.6p, and 2.6NRp strains. **(A)** Colony phenotypes of the recombinant and their parental strains as well as colony lifting of the β-glucan-producing strains. Visualization of bacteria by optical microscopy with an objective of 100×. **(B)** Overlays of phase contrast and fluorescence images of the four *P. parvulus* strains. Analysis of bacterial growth **(C)** and the red fluorescence emission **(D)** of the four *P. parvulus* strains measured with the Varioskan Flask System.

With regard to the expression of the mCherry fluorescent protein, *P. parvulus* 2.6p and 2.6NRp formed bright pink colonies on MRS-agar medium (**Figure 2A**), and this coloration was visible even after incubation of the plates for 16 days (Supplementary Figure S1). This long-term detection indicated the stability of mCherry labeling in these hosts. Moreover, the colonies of the 2.6p strain retained the ropy phenotype of its parental 2.6 strain (**Figure 2A**) indicating that both produce the 2-substituted (1,3)-β-D-glucan. To validate this hypothesis, supernatants of cultures of both pediococci were used to determine the concentration of EPS released to the medium by these strains, using an immunological method for specific detection of the pediococcal β-glucan. The results revealed a similar production of the EPS, e.g., at an OD_600 nm_ = 1.0, 114.97 ± 2.58 and 130.95 ± 6.53 mg L^-1^ for the 2.6 and 2.6p strains, respectively. In addition, expression of the mCherry protein was visualized at the cellular level by fluorescence microscopy. This analysis revealed an intense red color of the 2.6p and 2.6NRp bacteria, and superimposition of phase contrast and fluorescent images showed that all bacteria of both populations were expressing active mCherry protein (**Figure 2B**).

The use of the recombinant strains 2.6p and 2.6NRp instead of 2.6 and 2.6NR required that the genetic manipulation did not drastically affect the bacterial growth. Thus, the growth of the four *P. parvulus* strains in liquid medium was analyzed and the growth rate at the exponential phase was calculated (**Figure 2C**). The β-glucan-non-producing 2.6NR strain displayed a higher growth rate (μ = 0.231 ± 0.024) than that of the 2.6 strain (μ = 0.182 ± 0.005). The same behavior was observed, when the growth rate of the recombinant strains was compared μ = 0.184 ± 0.013 for 2.6NRp strain and μ = 0.174 ± 0.011 for 2.6p strain, but less differences between them were observed, a good characteristic for further comparative studies of bacteria–zebrafish interactions. In addition, the 2.6 and 2.6NR strains grew slightly faster than the corresponding recombinant strains. This behavior was expected, since only 2.6p and 2.6NRp were grown in the presence of Cm, because the pRCR12 plasmid confers resistance to this antibiotic. The high level of expression of the mCherry in the recombinant pediococci made it possible to analyze in real time, and simultaneously the growth (**Figure 2C**) and the fluorescence (**Figure 2D**) of the cultures. The levels of the red fluorescence emitted by the recombinant strains were increasing during the bacterial growth and remained stable during the stationary phase. These were similar in both strains and achieved a value of 111.27 ± 7.03 for 2.6NRp and of 93.18 ± 2.85 for 2.6p at OD_600nm_ = 1.0.

**FIGURE 3:**
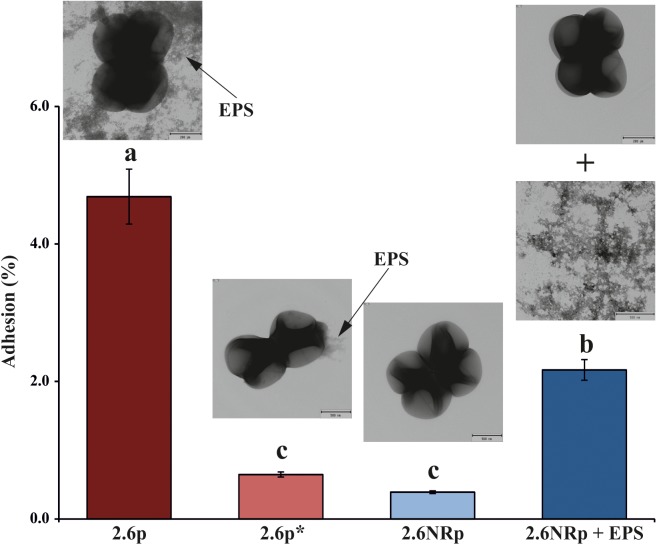
Bacterial adhesion to a monolayer of Caco-2 cells. Enterocytes were exposed independently to four bacterial solution containing: (i) 2.6p strain, (ii) 2.6p^∗^ treated to remove most of the β-glucan, (iii) 2.6NRp, and (iv) 2.6NRp plus the amount of purified β-glucan detected in 2.6p strain solution. The insets show electron micrographics of the bacteria. The arrows mark the β-glucan (EPS). Statistical significances are represented by different letters that mean *P* ≤ 0.01.

The capacity to adhere to the intestinal epithelium is one of the properties sought in probiotic bacteria. Therefore, we have previously investigated adhesion of *P. parvulus* 2.6 and 2.6NR strains to colon Caco-2 human cells (Fernández de Palencia et al., 2009), and detected a positive influence of the β-glucan in bacterial–human cells interaction. In this work, we had analyzed binding to these enterocytes of 2.6p with (called 2.6p^∗^) and without mechanical removal of its β-glucan and 2.6NRp alone or supplemented with *P. parvulus* 2.6 purified β-glucan (called 2.6NRp+EPS; **Figure 3**). In these types of experiments, prior to expose to the eukaryotic cell, the growth medium of the bacteria has to be removed by centrifugation. Thus, the β-glucan that remains attached to the bacteria after sedimentation and resuspension as well as after mechanical removal was quantified with the specific ELISA method. The results revealed that during the adhesion assay, the EPS bound to the 2.6p and 2.6p^∗^ samples, was at a concentration of 248 ± 11 and of 3.7 ± 0.1 ng mL^-1^, respectively. Moreover, as expected, the immunological assay revealed no detection of the β-glucan bound to 2.6NRp strain. Furthermore, analysis of the bacteria by transmission electronic microscopy confirmed the presence of the EPS bound to the 2.6p cells, the almost complete removal in the 2.6p^∗^, and its absence in 2.6NR (insets in **Figure 3**). The results of the binding assays showed that the 2.6p strain had a level of adhesion to Caco-2 cells significantly higher than that achieved by the EPS-non-producing strain 2.6NRp (4.7 *versus* 0.4%). These values of binding were similar to those of the parental 2.6 and 2.6NR strains (6.1 *versus* 0.2% and 4.7 *versus* 0.7%, respectively, in Fernández de Palencia et al. (2009) and in Supplementary Figure S2). Moreover, when the β-glucan of 2.6p was substantially reduced, the adherence of this bacterium was similar to that of the 2.6NRp strain (0.6 *versus* 0.4%). Conversely, when the β-glucan was added to the 2.6NRp strain, the adherence of this bacterium increased considerably (2.2 *versus* 0.4%), confirming that the β-glucan is directly involved in the adherence of *P. parvulus* to enterocytes. Therefore, the overall results demonstrate the suitability of the 2.6p and 2.6NRp strains, rather than the parental strains to perform validated *in vivo* studies of zebrafish larvae–pediococci interactions.

### *In Vivo* Evaluation of Probiotic Properties of *P. parvulus* 2.6p and 2.6NRp Strains in Zebrafish Model

**FIGURE 4:**
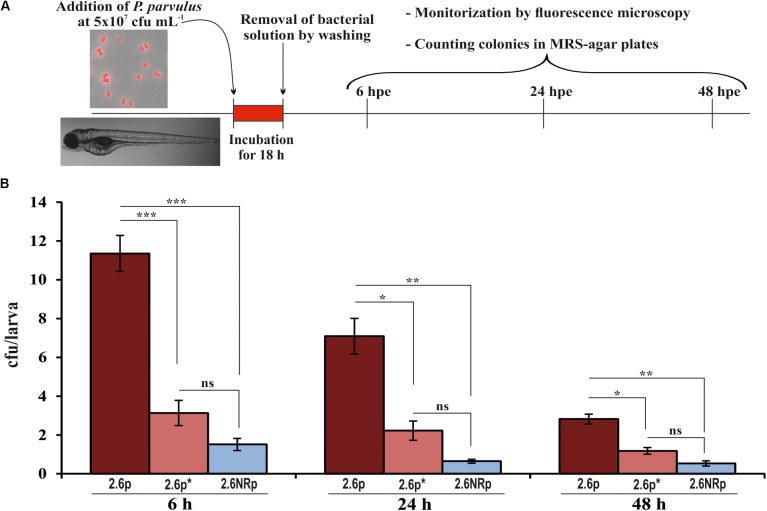
Zebrafish gut colonization by *P. parvulus* 2.6p and 2.6NRp strains. **(A)** The experimental procedure is depicted. Four-days-old larvae were colonized during 18 h by 2.6p with (2.6p^∗^) or without β-glucan removal or 2.6NRp strains. **(B)** The pediococcal colonies presented inside the larvae were analyzed at 6, 24, and 48 h after the bacteria were removed from the incubation buffer (hours post exposure, hpe). Differences between conditions were evaluated at each point time. Statistical significances are represented by ^∗^*P* ≤ 0.05, ^∗∗^*P* ≤ 0.01, ^∗∗∗^*P* ≤ 0.001, and ns (*P* > 0.05).

**FIGURE 5:**
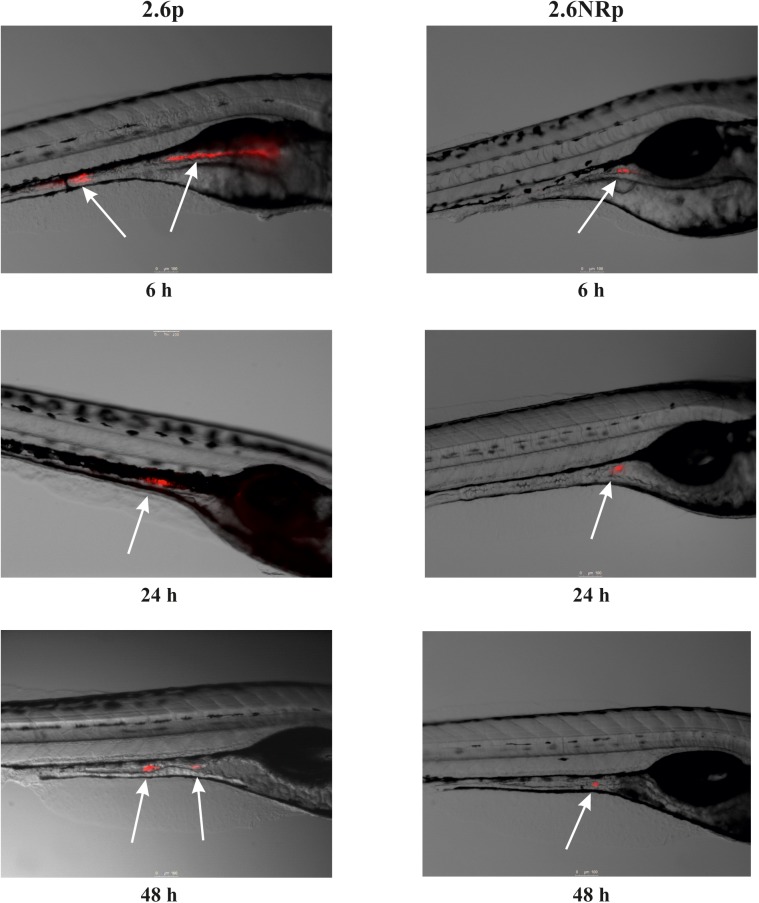
Fluorescence microscopy images of zebrafish gut colonization by *P. parvulus* 2.6p and 2.6NRp strains. Representative images taken at 6, 24, and 48 hpe are depicted. White arrows mark the red fluorescence signal emitted by the bacterial cells inside the zebrafish gut.

Animal models are necessary to validate the probiotic properties of bacteria. Currently, the zebrafish models are used for this purpose (Rendueles et al., 2012; Russo et al., 2015; Nácher-Vázquez et al., 2017). Thus, the colonization capacity of the 2.6p and 2.6NRp strains was tested by their exposure to gnotobiotic zebrafish larvae and quantified by plating the bacteria present in the intestinal tract of the larvae according to the protocol depicted in **Figure 4A**. For the two strains and in all conditions tested, the prevalence in the zebrafish gut decreased with the time of incubation (**Figure 4B**). In addition, at all the times tested (6, 24, and 48 hpe to the bacteria), the results corroborated *in vivo* the positive influence of the β-glucan in adherence capability of *P. parvulus* 2.6p. The colonization by this bacterium was significantly higher than that of the EPS-non-producing strain 2.6NRp (11.4, 7.1, and 2.8% *versus* 1.5, 0.6, and 0.5% at 6, 24, and 72 hpe, respectively). In addition, removal of the β-glucan decreased the colonization levels of the 2.6p strain to values (3.1, 2.2, and 1.2% at 6, 24, and 72 hpe, respectively) no significantly different to that of the 2.6NRp. Furthermore, these results correlated with those obtained in the bacteria–Caco-2 cells’ interaction studies (**Figure 3**). Moreover, the mCherry-labeling of the *P. parvulus* strains allowed monitoring the zebrafish gut colonization by fluorescence microscopy. In **Figure 5**, are depicted representative images of 2.6p and 2.6NRp strains on larvae intestinal epithelium. In both cases, the levels of fluorescence decreased with the incubation time and they were higher in the case of 2.6p.

**FIGURE 6:**
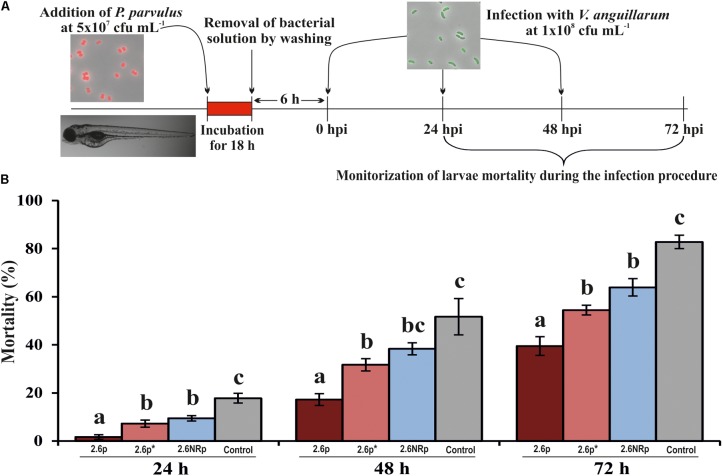
Protective effect of *P. parvulus* 2.6p and 2.6NRp strains against *V. anguillarum* NB10[pOT11] zebrafish infection. **(A)** The experimental procedure. Before infection with *V. angillarum*, 4-days-old larvae were colonized during 18 h either by 2.6p with (2.6p^∗^) or without β-glucan removal or by 2.6NRp strains. Also, as a control, a group of larvae was incubated in the absence of bacteria during 18 h prior infection. **(B)** Cumulative mortality due to *Vibrio* infection was analyzed at 24, 48, and 72 h after addition of the pathogen (hours post infection, hpi). Differences between conditions were evaluated at each point time. Statistical significances are represented by different letters that mean *P* ≤ 0.05.

Also, the influence of *P. parvulus* colonization against infection by *V. anguillarum* was investigated. To this end, gnotobiotic zebrafish larvae previously colonized by either *P. parvulus* 2.6p, 2.6p^∗^, or 2.6NRp strains were infected with *V. anguillarum* NB10[pOT11] according to the protocol depicted in **Figure 6A**. The results showed 100% of larvae survival in the absence of treatment with bacteria (data not shown). In addition, they revealed that both 2.6p and 2.6NRp strains were able to decrease significantly the larvae mortality due to *Vibrio* infection, being more pronounced the effect of 2.6p without EPS removal (**Figure 6B**). The protective effect was extended during the entire infection assay (**Figure 6B**). Thus, at 72 hpi, a survival of only 17.2% was detected in the control group infected with *V. anguillarum* and not pre-treated with LAB, whereas in the 2.6p-colonized group, a 60.6% of alive larvae was observed. Furthermore, the groups colonized by 2.6p^∗^ and 2.6NRp achieved an intermediate degree of survival, 45.5 and 36.1%, respectively. Therefore, colonization by the LAB seems to play a role in the inhibition of pathogenic effect of *V. anguillarum* NB10[pOT11]. Thus, the overall results support the potential use of *P. parvulus* 2.6 as probiotic to prevent fish infections.

### Immunomodulatory Properties of the 2-Substituted (1,3)-β-D-Glucan in Zebrafish Models

The zebrafish is currently used for immunomodulation studies, since its innate immunological response is similar to that of other vertebrates (Novoa and Figueras, 2012; de Oliveira et al., 2013). Thus, the purified β-glucan of *P. parvulus* 2.6 could be evaluated as an immunomodulator in zebrafish models.

**FIGURE 7:**
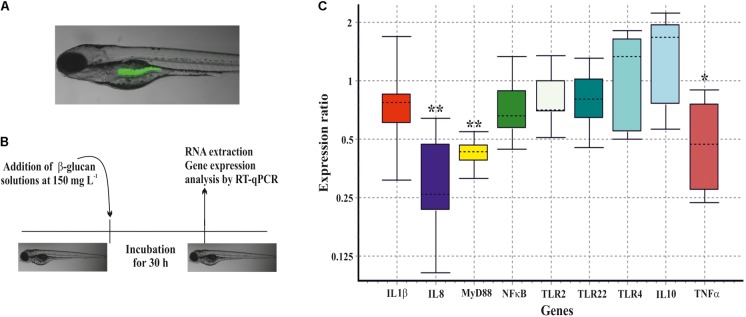
Immunomodulation of gnotobiotic zebrafish larvae by the *P. parvulus* 2.6 β-glucan. **(A)** DTAF-labeled β-glucan was detected inside the zebrafish gut. **(B)** The experimental procedure for the immunomodulation assay. **(C)** Relative expression values of the genes involved in inflammation. The genes actin-β and elongation factor-1 were used as housekeeping to normalize the values. Statistical significances are represented by ^∗^*P* ≤ 0.05 and ^∗∗^ *P* ≤ 0.01.

Prior to this evaluation, the capability of gnotobiotic zebrafish larvae to ingest the β-glucan was tested. The purified EPS was green-fluorescently-labeled with DTAF and, 6 h after exposure, fluorescence was detected in the zebrafish gut by fluorescence microscopy (**Figure 7A**). Afterward, the unlabeled *P. parvulus* 2.6 β-glucan was evaluated in two zebrafish models. First, it was studied its effect on the immune system of non-stimulated zebrafish larvae. Gnotobiotic larvae at 4 dpf were submerged in β-glucan solutions at 150 μg mL^-1^ for 30 h, after that variation on gene expression of nine immune related genes was evaluated (**Figure 7C**) according with the protocol depicted in **Figure 7B**. The exposure of larvae to the β-glucan caused the downregulation of three genes involved in inflammatory response. Expression of two pro-inflammatory cytokine genes, IL8 and TNFα, was repressed 3.4-fold and 2.2-fold, respectively, and the adapter protein gene MyD88 was repressed 2.4-fold. In addition, the expression of anti-inflammatory cytokine gene IL10 showed a non-significant tendency to increase. The rest of the genes evaluated did not show any change in their expression levels.

**FIGURE 8:**
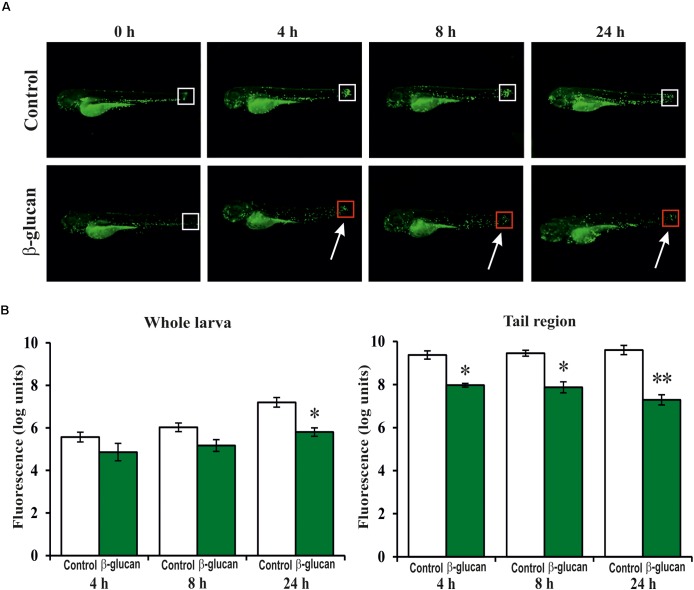
Anti-inflammatory effect of the *P. parvulus* 2.6 β-glucan in an induced inflammation model of the *Tg*(*mpx:GFP*) zebrafish line. The inflammation was induced by cutting off the apical region of the tail. **(A)** Images of representative larvae treated and untreated with the β-glucan are depicted. Squares mark the areas of inflammation, and the colors indicate regions (red) with less migration of neutrophils than others (white). **(B)** The green fluorescence emitted by the neutrophils was quantified in the whole larvae and in their tails regions. The fluorescence values detected during the inflammation procedure were expressed as increased percentages of the values detected at 0 h, and represented as logarithms. Statistical significances between groups are represented by ^∗^*P* ≤ 0.05 and ^∗∗^*P* ≤ 0.01.

In addition, the β-glucan was evaluated in an induced inflammation model using the zebrafish transgenic line *Tg*(*mpx:GFP*)*i114*. In this line, the neutrophils are GFP-labeled, allowing their detection during the inflammation response. The inflammation in the larvae was induced by cutting the apical region of the tail. Then, the recruitment and proliferation of the neutrophils in the larvae, during the first 24 h of the inflammation process, was visualized (**Figure 8A**) and quantified (**Figure 8B**) by fluorescence microscopy and images analysis, respectively. Quantification of the GFP levels at the tail region revealed that in the control larvae group, not exposed to the β-glucan, the recruitment of the neutrophils in the inflammation area drastically increased (9.4 logs) during the first 4 h and remained at this level (9.6 log) even after 24 h. However, the larvae treated with the β-glucan showed a lower recruitment of neutrophils with an increase of 8.0 logs during the first 4 h, that was further reduced to 7.3 logs after 24 h. Attending to the whole fluorescence signal in larvae, what is representative of neutrophil proliferation, there were also differences between the control and the treated larvae groups after 24 h, when the fluorescence increases were 7.2 and 5.8 logs, respectively. These results indicated that in zebrafish larvae subjected to an inflammatory process, the presence of the β-glucan provoked an anti-inflammatory response manifested by a decreasing of the recruitment and proliferation of the neutrophils.

## Discussion

Disease control and prevention strategies in aquaculture have been based primarily on the use of antibiotics. However, this indiscriminate use can lead to major drawbacks such as the emergence of resistant strains, contamination of the aquatic environment, toxicity to the organism, and the accumulation of residues in organs and tissues, as well as other potential negative effects on human health. For this reason, it is necessary to drastically reduce the use of antibiotics in aquaculture (Cabello, 2006; Defoirdt et al., 2011). On the other hand, the normal microbiota of the digestive tract of fish plays a fundamental role in the exclusion of pathogens and in the maintenance of health. In this way, the use of probiotic bacteria (Newaj-Fyzul et al., 2014; Hai, 2015) can improve the feed conversion efficiency and live weight gain. In addition, probiotics can confer protection against pathogens by competitive exclusion for adhesion sites, production of organic acids (formic acid, acetic acid, and lactic acid), hydrogen peroxide, bacteriocins, or lysozyme and also modulate physiological and immunological responses in fish (Nayak, 2010). Thus, the use of probiotic bacteria is another strategy for improving production in fish farms.

Consequently, in this work, we have characterized the probiotic properties of the 2-substituted (1,3)-β-D-glucan-producing *P. parvulus* 2.6 in comparison with the non-producing *P. parvulus* 2.6NR strain to evaluate its potential as a probiotic in aquaculture, upon mCherry-labeling by transfer of pRCR12 plasmid. There are a very few works reporting electrotransformation of strains belonging to the *Pediococcus* genus, e.g., transfer of derivatives of pRS4 plasmid of *Pediococcus pentosaceus* (Alegre et al., 2005) by electroporation to *Pediococcus acidilactici* (Rodríguez et al., 2007). In addition, as far as we know this is the first report of plasmid transfer to and fluorescence-labeling of *P. parvulus* strains, which did not significantly affect bacterial growth and β-glucan production.

Prior having a beneficial effect in the digestive tract, probiotic bacteria must resist among others to the stress due to the digestive enzymes. Previously, the survival of *P. parvulus* 2.6 and 2.6NR strains under an *in vitro* gastrointestinal model system was demonstrated (Fernández de Palencia et al., 2009), but no influence of the β-glucan in resistance to the gut stresses was observed. However, when the *P. parvulus* 2.6 β-glucan was expressed in *Lactobacillus paracasei*, the presence of this EPS increased the bacterial survival to gastrointestinal stresses (Stack et al., 2010). In addition, the same effect of the polymer was observed, when the survival of *P. parvulus* 2.6 and 2.6NR strains through the digestive tract was evaluated using an *in vivo* mice model (Lindström et al., 2012). Accordingly, the results presented here revealed that *P. parvulus* 2.6 and 2.6NR remained viable inside the zebrafish gut, and this viability was improved in the presence of the β-glucan.

Many of the health effects exerted by the probiotic bacteria are related to their capability to adhere to the intestinal cells. Therefore, this is one of the main criteria for the selection of probiotic strains. In general, the scientific reports support that synthesis of EPS by LAB decreases the adhesion ability of the producing bacteria to enterocytes. This is the case of heteropolysaccharides of *Lactobacillus rhamnosus* (Lebeer et al., 2009; Polak-Berecka et al., 2014) and *Lactobacillus johnsonii* (Horn et al., 2013; Dertli et al., 2015) as well as dextrans (α-glucan homopolysaccharides) produced by *L. sakei* and *Leuconostoc mesenteroides* strains (Nácher-Vázquez et al., 2017; Zarour et al., 2017). However, Živković et al. (2016) detected a positive influence of an heteropolysaccharide of *L. paracasei* in bacterial adhesion to epithelial intestinal cells. Furthermore, we have demonstrated *in vitro* that the presence of the 2-substituted (1,3)-β-D-glucan promotes adhesion of *P. parvulus* strains to enterocytes (Fernández de Palencia et al., 2009; Garai-Ibabe et al., 2010; and this work). Moreover, the results presented here support that the presence of this EPS enhances *in vivo* the capabilities of *P. parvulus* for colonization of the zebrafish intestinal tract, by contrast with the dextran from *L. sakei*, which diminish the stay of the bacteria in the fish gut (Nácher-Vázquez et al., 2017). Also, the results obtained in this work suggest a transient colonization of the zebrafish gut by *P. parvulus* 2.6 strain. This behavior is expected for a probiotic bacteria, that by definition should be administrated daily (Guarner et al., 2017), and it has been previously detected with this zebrafish model for *L. plantarum*, *L. fermentum*, and *L. sakei* probiotic strains (Russo et al., 2015; Nácher-Vázquez et al., 2017).

Other property described for probiotic bacteria is to compete with pathogens involved in infection diseases, which are frequent in fish farms. Thus, in the last few years, there have been several reports concerning to the beneficial effect of probiotic candidates against various bacterial infections (Ran et al., 2012; Saini et al., 2014; Mohideen and Haniffa, 2015). Among the fish pathogens, *V. anguillarum* causes a deadly hemorrhagic septicemia disease named vibriosis, which provokes high morbidity and mortality rates and it is responsible for severe economic losses (Frans et al., 2011). In this context, we have previously showed that *L. sakei* MN1 competes with *V. anguillarum* in the zebrafish digestive tract (Nácher-Vázquez et al., 2017) and the results presented here revealed that pre-treatment with *P. parvulus* 2.6 has even a higher protective effect that *L. sakei* against vibriosis. Competition of probiotic with pathogenic bacteria could be due to either displacement, blocking their interaction with the epithelium, or stimulation of the immune system. In the case of *P. parvulus*, our results demonstrated that its β-glucan significantly contributes to its ability to compete with *V. anguillarum*, presumably by blocking the interaction of the pathogen with the enterocytes, and not by its displacement, since the zebrafish larvae were pre-treated with the LAB. However, the results presented here also suggest that the immunomodulation provoked by the *P. parvulus* 2.6 β-glucan (see details below) could contribute to the protective effect of the bacteria against *V. anguillarum* infection. Moreover, a killing of the pathogen by *P. parvulus* due to the LAB synthesis of antimicrobial compounds could take place, and in fact, there was *in vitro* detection of growth inhibition of a *V. anguillarum* lawn, when the pathogen was exposed to *P. parvulus* 2.6, 2.6p, 2.6NR, or 2.6NRp strains (Supplementary Figure S3A). This effect is presumably due to lactic acid secreted by the LAB, since exposure of the pathogen to the acidic (pH 4.0) bacterial culture supernatants yielded a small inhibition halo, which was not detected after the supernatants neutralization to pH 6.5 (Supplementary Figures S3B,C). However, this does not seem to be the major cause of the anti-infective activity of *P. parvulus* 2.6, because the same inhibition pattern was observed with the four strain tested. Nevertheless, independently of the mechanisms involved in the performance of *P. parvulus* 2.6, the studies of bacteria–zebrafish interactions performed in this work support the potential utilization of this bacterium as a probiotic in aquaculture.

Intensive fish farming causes animals to be subjected to stress conditions that weaken their immune system, increasing susceptibility to pathogens and thus promoting the emergence of diseases. Therefore, to prevent and control infections, the immunostimulants are currently utilized as feed additives, as an alternative approach to control these problems (Ringø et al., 2012). The most widely used is the β-glucan with (1,3)-linkages from *Saccharomyces cerevisiae* yeast cell wall (Immunogen^®^ and A-Max are commercialized products) containing β-glucans and mannanoligosaccharides (Yar Ahmadi et al., 2014; Akrami et al., 2015). These products have a positive effect on innate immune parameters, growth, feed efficiency, and resistance against *Aeromonas hydrophila* of rainbow trout. In this work, the exposure of gnotobiotic zebrafish larvae to the *P. parvulus* 2.6 β-glucan produced the inhibition of gene expression of two pro-inflammatory cytokines, TNFα and IL8. In addition, the protein adaptor MyD88, which mediates activation of pro-inflammatory cytokines via NF-κB, was inhibited. TNFα is one of the most important cytokines involved in inflammation responses (Zelová and Hošek, 2013). On the other hand, IL8 (also called CXCL8) is a member of the chemokines family, which facilitates in immune cells (mainly neutrophils) their migration, accumulation, and activity at the inflammation sites (de Oliveira et al., 2013). The neutrophil recruitment is mediated by two G-protein coupled receptors, CXCR1 and CXCR2 (Oehlers et al., 2010), and the use of an induced inflammation model in the *Tg*(*mpx:GFP*) zebrafish line has allowed us to study the behavior of the neutrophils in the presence of the *P. parvulus* 2.6 β-glucan. The results showed that the recruitment and proliferation of the neutrophils in the larvae exposed to the β-glucan were inhibited. Thus, these results support that the 2-substituted (1,3)-β-D-glucan specifically affects the signaling pathway of IL8 producing an anti-inflammatory response, corroborating the anti-inflammatory activity of this polymer on human macrophages (Notararigo et al., 2014). However, Lindström et al. (2012) showed that the purified *P. parvulus* 2.6 β-glucan did not produce any anti-inflammatory response in an *in vivo* mice model, and this discrepancy could be due to differences in the procedures for isolation and purification of the EPS. Nevertheless, in the mice model was detected a higher pro-inflammatory response to 2.6NR than to 2.6 strains (Lindström et al., 2012). Thus, this last result correlates with our previous findings showing differential *in vitro* immunomodulation of human macrophages by these two strains (Fernández de Palencia et al., 2009). In addition, they support that the natural presence of the β-glucan attached to the cell wall of 2.6 strain counteracts the pro-inflammatory effect of *P. parvulus*.

Finally, the current problems in aquaculture indicate that a combination of polysaccharides with immunomodulatory capacity and a probiotic bacterium could be of interest for the improvement of fish production, since they can be utilized to generate functional symbiotic feed for use in fish farms (Huynh et al., 2017). In this context, the study presented here supports the use of *P. parvulus* 2.6 as a probiotic producing *in situ* its β-glucan for the elaboration of fish feed with potential anti-inflammatory and anti-pathogenic properties.

## Ethics Statement

AZTI-Tecnalia has all legal authorization for housing animals, breeding animals, and performing experiments with animals in Spain (animal facility registration number EU-10-BI and REGA code ES489010006105). Dr. Miguel Angel Pardo is responsible of zebrafish facilities and taking part in one of the National Animal Welfare Bodies (Former Ethic Committee), which is accredited for performing animal experiments to the required National and European legislative demands (Council Directive 2010/63/EU), within the project ENVIPHAGE, NEIKER-OEBA-2015-004.

## Author Contributions

AP-R contributed to all parts of the experimental work and wrote a draft of the manuscript. MP contributed to the design and analysis of the experimental work involving zebrafish models. MM contributed to the design of strategies to develop and analyze the bacterial recombinant strains and corrected the manuscript. PL participated in study conception and data interpretation and generated the final version of the manuscript. All authors read and approved the final manuscript.

## Conflict of Interest Statement

The authors declare that the research was conducted in the absence of any commercial or financial relationships that could be construed as a potential conflict of interest. The handling Editor declared a past co-authorship with several of the authors (AP-R, MM, and PL).
