# Supplementary material for: β-Glucan-Producing Pediococcus parvulus 2.6: Test of Probiotic and Immunomodulatory Properties in Zebrafish Models
Source: Front Microbiol. 2018 Jul 25;9:1684. doi: 10.3389/fmicb.2018.01684 (PMC6068264; doi:10.3389/fmicb.2018.01684)
Supplement: Supplementary file 1 [file Data_Sheet_1.PDF]

Supplementary material

β-glucan-producing *Pediococcus parvulus* 2.6: test of probiotic and immunomodulatory properties in zebrafish models

*Pérez-Ramos A.[1], Mohedano M.L.[1], Pardo M.A.[2], López P.[1]**

[1]Laboratory of Molecular Biology of Gram-positive Bacteria. Department of Microorganisms and Plant Biotechnology. Biological Research Center (CIB), CSIC, Madrid, Spain
[2]Food Research Division, AZTI-Tecnalia, Bizkaia, Spain

*** Correspondence:**

Paloma López
plg@cib.csic.es

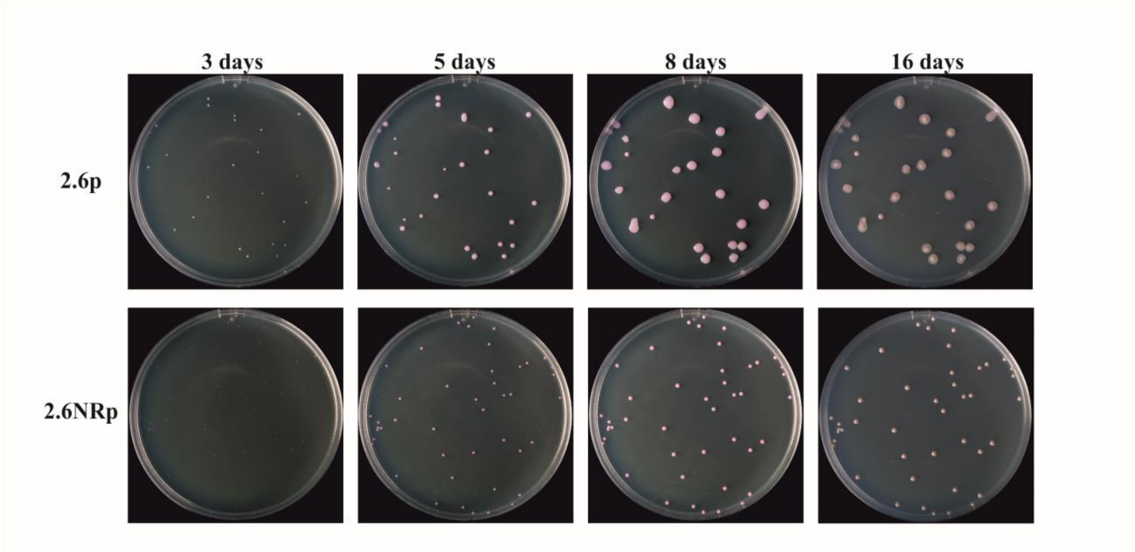

Figure S1. Detection of mCherry production by *P. parvulus* 2.6[pRCR12] (2.6p) and *P. parvulus* 2.6NR[pRCR12] (2.6NRp) on solid media. Bacterial colonies were grown in MRS plates during 16 days. Images of plates at 3 days, 5 days, 8 days and 16 days are depicted.

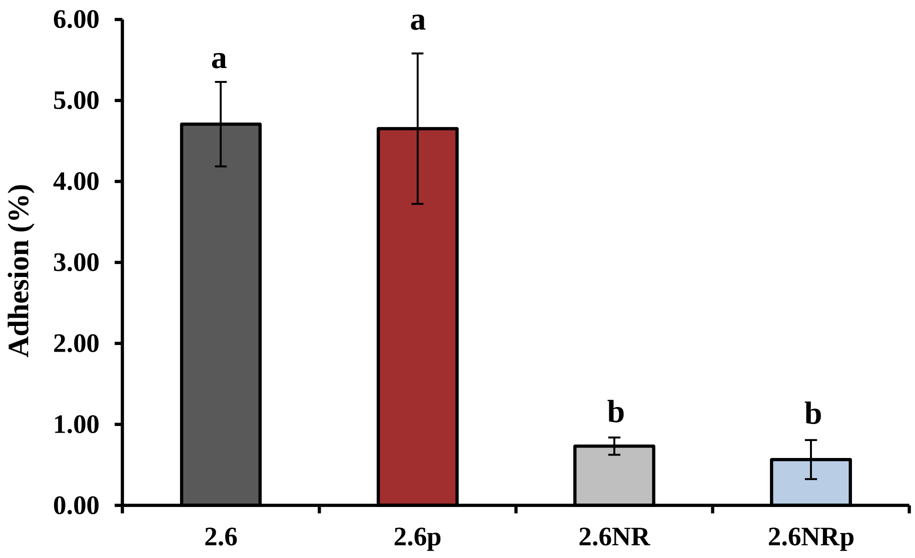

Figure S2. *P. parvulus* adhesion to a monolayer of Caco-2 cells. Enterocytes were exposed independently to four bacterial solution containing 2.6, 2.6[pRCR12] (2.6p). 2.6NR or 2.6NR[pRCR12] (2.6NRp) strains. Statistical significances are represented by different letters that mean a P≤0.01.

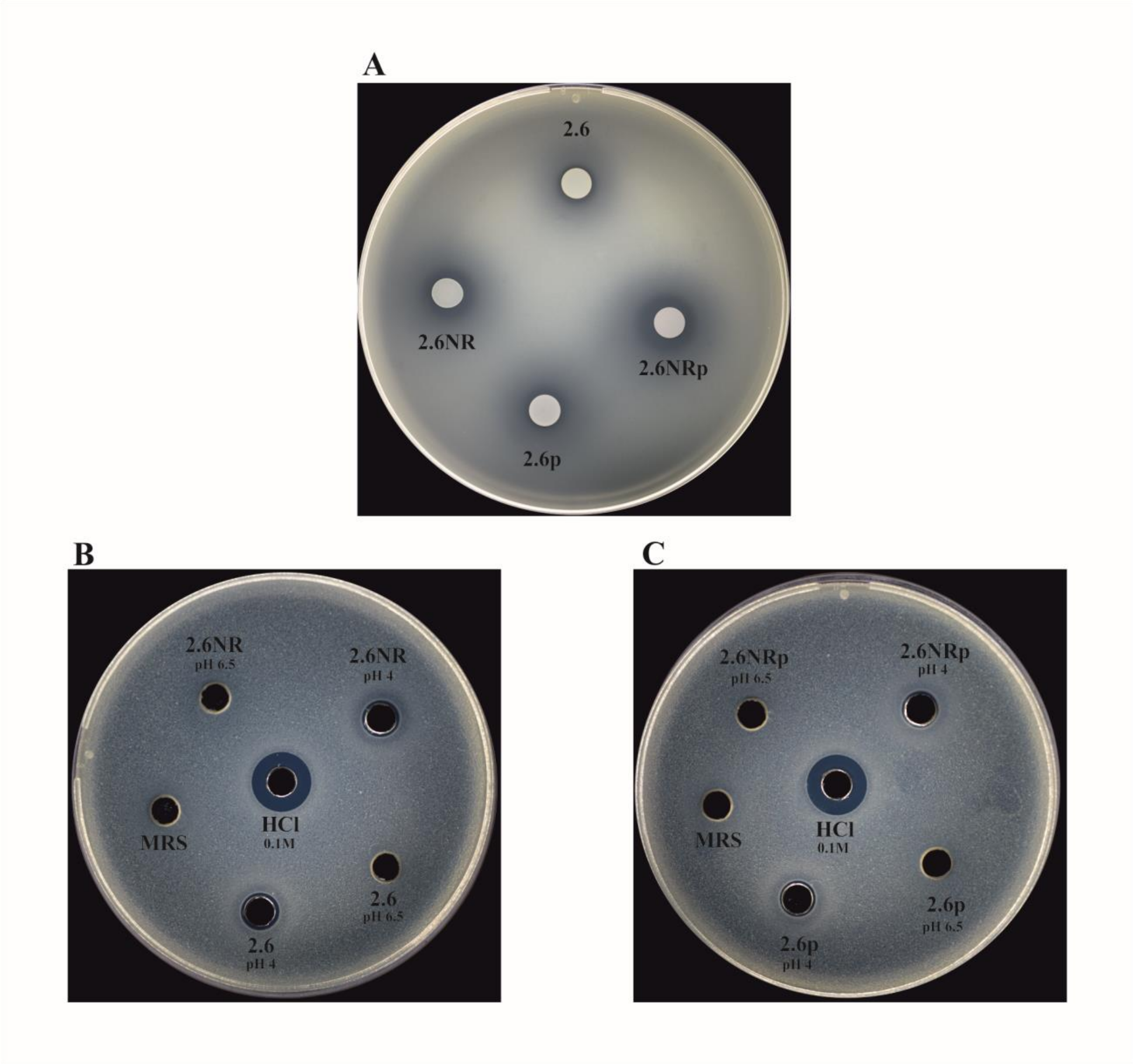

Figure S3. Antimicrobial activity of *P. parvulus* strains against *V. anguillarum*. The activity of spots of the four indicated strains was tested **(A).** A diffusion test was performed with the supernatants of stationary phase cultures of either 2.6 or 2.6NR **(B)** and either 2.6p or 2.6NRp **(C)**. Samples of supernatants with pH 4.0 were neutralized to pH 6.5, and both samples were tested. 0.1 M HCl, and MRS medium were used as a positive and a negative controls.

Table S1. Antibiotic resistant profile of *P. parvulus* 2.6 and 2.6NR strains.

Antibiotic	Concentrations tested (mg L^{-1})	2.6	2.6NR
Ampicillin	1	+	+
	2	+	+
	4*	-**	-**
	8	-	-
Gentamycin	4	-**	-**
	8	-	-
	16*	-	-
	32	-	-
Kanamycin	16	+	+
	32	-**	-**
	64*	-	-
	128	-	-
Streptomycin	16	-**	-**
	32	-	-
	64*	-	-
	128	-	-
Erythromycin	0.25	-**	-**
	0.5	-	-
	1*	-	-
	2	-	-
Clindamycin	0.25	-**	-**
	0.5	-	-
	1*	-	-
	2	-	-
Tetracycline	2	+	+
	4	-**	-**
	8*	-	-
	16	-	-
Chloramphenicol	1	+	+
	2	-**	-**
	4*	-	-
	8	-	-

*Microbiological cut-off values according to EFSA (2012).

**MIC: minimal inhibitory concentration.
